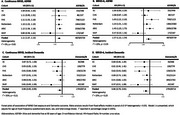# Higher MIND diet scores are associated with decreased all‐cause dementia risk and being alive and dementia free at age 80: the cross‐cohort collaboration

**DOI:** 10.1002/alz70860_098370

**Published:** 2025-12-23

**Authors:** Debora Melo van Lent, Daniel Koijs, Josh Bis, Robin Bülow, Tosca O.E. de Crom, Carole Dufouil, Hans J. Grabe, Leslie Grasset, Monica Goss, Stefan Frenzel, Jayandra Jung Himali, Arfan Ikram, Oscar L Lopez, Thomas H. Mosley, Cécilia Samieri, Claudia L Satizabal, Jeannette Simino, Aline Thomas, Henry Voelzke, Trudy Voortman, Frank J. Wolters, Amber Yaqub, Sudha Seshadri, Alexa S Beiser

**Affiliations:** ^1^ Framingham Heart study, Framingham, MA, USA; ^2^ Glenn Biggs Institute for Alzheimer's & Neurodegenerative Diseases, University of Texas Health San Antonio, San Antonio, TX, USA; ^3^ The Framingham Heart Study, Framingham, MA, USA; ^4^ Department of Biostatistics, Boston University School of Public Health, Boston, MA, USA; ^5^ Boston University School of Public Health, Boston, MA, USA; ^6^ University of Washington, Seattle, WA, USA; ^7^ University Medicine Greifswald, Greifswald, Mecklenburg‐Voor‐Pommeren, Germany; ^8^ Erasmus University Medical Center, Rotterdam, Zuid Holland, Netherlands; ^9^ Centre INSERM U1219, Institut de Santé Publique, d'Epidémiologie et de Développement (ISPED), Bordeaux School of Public Health, Université de Bordeaux, Bordeaux, France; ^10^ University Medicine Greifswald, Greifswald, Germany; ^11^ Centre INSERM U1219, Institut de Santé Publique, d'Epidémiologie et de Développement (ISPED), Bordeaux School of Public Health, Université de Bordeaux, Bordeaux, FL, France; ^12^ Glenn Biggs Institute for Alzheimer's & Neurodegenerative Diseases, University of Texas Health Science Center, San Antonio, TX, USA; ^13^ Department of Population Health Sciences, University of Texas Health Sciences Center, San Antonio, TX, USA; ^14^ Glenn Biggs Institute for Alzheimer's & Neurodegenerative Diseases, University of Texas Health San Antonio, San Antonio, TX, USA; ^15^ Department of Neurology, Boston University Chobanian & Avedisian School of Medicine, Boston, MA, USA; ^16^ Erasmus University Medical Center, Rotterdam, South Holland, Netherlands; ^17^ University of Pittsburgh Alzheimer's Disease Research Center (ADRC), Pittsburgh, PA, USA; ^18^ University of Mississippi Medical Center, The MIND Center, Jackson, MS, USA; ^19^ Bordeaux Population Health Research Center, Inserm U1219, University of Bordeaux, Bordeaux, NA, France; ^20^ Framingham Heart Study, NHLBI, Framingham, MA, USA; ^21^ The University of Texas Health Science Center at San Antonio, San Antonio, TX, USA; ^22^ University of Mississippi Medical Center, Jackson, MS, USA; ^23^ Taub Institute for Research on Alzheimer's Disease and the Aging Brain, Columbia University, New York, NY, USA; ^24^ Univ. Bordeaux, Inserm, BPH, U1219, Bordeaux, France; ^25^ Erasmus University Medical Center, Rotterdam, Netherlands; ^26^ Erasmus MC ‐ University Medical Center Rotterdam, Rotterdam, Zuid‐Holland, Netherlands; ^27^ Department of Epidemiology, Erasmus University Medical Center, Rotterdam, Zuid Holland, Netherlands; ^28^ Glenn Biggs Institute for Alzheimer's & Neurodegenerative Diseases, University of Texas Health Sciences Center at San Antonio, San Antonio, TX, USA; ^29^ South Texas Alzheimer's Disease Research Center, San Antonio, TX, USA; ^30^ Boston University and the NHLBI's Framingham Heart Study, Boston, MA, USA; ^31^ Boston University Chobanian & Avedisian School of Medicine, Boston, MA, USA

## Abstract

**Background:**

Findings from observational studies which examined relationships between Mediterranean‐ DASH for Neurodegenerative Delay(MIND) diet scores and risk of dementia are promising. We investigated whether MIND diet scores are associated with incident all‐cause dementia and being alive and dementia free at age 80 across six community‐based cohorts.

**Method:**

We analyzed data from 8714 dementia free adults (mean age range across cohorts: 67 to 73) who participated in the Atherosclerosis Risk in Communities (ARIC) cohort, Cardiovascular Health Study (CHS), Three City (3C) cohort, Framingham Offspring Study (FOS) cohort, Rotterdam Study (RS) or the Study of Health in Pomerania (SHIP) cohort. Individuals had incident all‐cause dementia surveillance data available and completed a validated food frequency questionnaire (FFQ) (ARIC, CHS, FOS, RS, 3C), 24h dietary recall (3C), or an extensive food list (SHIP). The MIND diet score is derived from ten healthy and five unhealthy components. We used Cox regression and logistic regression. Results were combined in meta‐analysis using fixed effects and random effects models.

**Result:**

Higher MIND diet scores (score range: 0‐15) and MIND diet scores ≥6 were associated with decreased risk for all‐cause dementia (Hazard ratio(HR) = 0.96,95% confidence interval(CI)=0.93‐0.99; 0.86,95%CI=0.78‐0.94, respectively) after adjustment for age at dietary intake assessment visit, sex, and total energy intake. Further, higher MIND diet scores and MIND diet scores ≥6 related to higher odds of being alive and dementia free at age 80 (Odds ratio(OR) = 1.11,95%CI=1.07‐1.15;OR=1.37,95%CI=1.22‐1.54, respectively). Heterogeneity between studies ranged between 0‐30%.

**Conclusion:**

Higher MIND diet scores associated with decreased all‐cause dementia risk and being alive and dementia free at age 80. We encourage clinical trials to examine the MIND diet in relation to dementia related outcomes such as amyloid and tau to elucidate whether a causal relationship between the MIND diet and dementia pathologies exists.